# Outbreak of carbofuran and bifenthrin poisoning in siblings after ingestion of contaminated food in KSA: Clinical management and public health implications

**DOI:** 10.1016/j.jtumed.2024.12.012

**Published:** 2025-01-18

**Authors:** Musa S. Alfaifi, Abrar K. Alasmari, Abdullah M. Assiri, Bader A. Alyahya, Nafaa A. Abdulalem, Hussein A. Albogami, Hamoud M. Alrougi, Adel M. Altowairqi, Rayyan M. Saqah, Maged A. Hamoud, Hajer F. Ali, Zohair A. Al Aseri

**Affiliations:** a Department of Emergency Medicine, Armed Forces Hospital Southern Region, Khamis Mushait, KSA; b Assistance Agency for Preventive Health, Saudi Ministry of Health, Riyadh, KSA; c Department of Emergency Medicine, College of Medicine, King Saud University, Riyadh, KSA; d Department of ICU, Ahd Rofidah General Hospital, Kamis Mushayt, KSA; e Public Health Agency, General Directorate of Environmental Health, Ministry of Health, KSA; f Emergency Medicine Department, Alhada Armed Force Gospital, Taif, KSA; g Pediatric Department, Maternity & Children's Hospital, Bisha, KSA; h Department of Clinical Sciences, College of Medicine and Riyadh Hospital, Dar Al Uloom University, Riyadh, KSA; i Depatments of Emergency Medicine and Critical Care, College of Medicine, King Saud University, Riyadh, KSA; j Adult Critical Care, Therapeutic Deputyship, Ministry of Health, KSA

**Keywords:** التسمم بالمبيدات الحشرية, الكاربارمات, البيريثرويد, التسمم الحاد, الأعراض الكولينية, المملكة العربية السعودية, Acute poisoning, Carbamates, Cholinergic symptoms, KSA, Pesticide poisoning, Pyrethroid

## Abstract

**Background:**

Pesticide poisoning remains a major global health concern contributing to an estimated 371,594 deaths annually. Both accidental and deliberate pesticide exposure, particularly in developing countries, pose challenges to public health systems. In KSA, pesticide poisoning is prevalent, and insecticides such as carbamates and organophosphates are the primary culprits.

**Case presentation:**

Four cases of acute poisoning caused by the ingestion of contaminated shawarma sandwiches containing carbofuran and bifenthrin, a carbamate and pyrethroid mixture, respectively, are described. The affected individuals, all siblings, exhibited varying degrees of cholinergic symptoms, including muscle fasciculations, excessive secretions, and respiratory distress. Despite their severe symptoms, three patients had normal or low-normal acetylcholinesterase levels, probably because of individual variability, delayed testing, or improper sample handling. One patient exhibited diminished acetylcholinesterase activity, thus suggesting severe inhibition; pancreatitis was a further complication that contributed to delayed recovery. Early clinical management included atropine, pralidoxime, and botulinum antitoxin, because of initial suspicion for botulism. Toxicological analysis confirmed carbofuran poisoning, and food safety investigations identified improper pesticide use in food preparation areas.

**Conclusion:**

The reported cases underscore the importance of timely toxicological consultation, proper testing, and appropriate sample handling to guide treatment of pesticide-related poisoning. These findings highlight the crucial need for adherence to WHO regulations and monitoring of pesticide use, as well as strict food safety practices to prevent future outbreaks. The successful multidisciplinary approach to managing these cases demonstrates the critical role of coordinated efforts between clinical teams and public health authorities in addressing such public health threats.

## الملخص

****المقدمة**:**

يظل التسمم بالمبيدات الحشرية مشكلة صحية عامة كبيرة على مستوى العالم، مما يسهم في حوالي 371,594 حالة وفاة سنويًا. يتسبب التعرض العرضي والمتعمد للمبيدات الحشرية، خاصة في الدول النامية، في تحديات كبيرة لأنظمة الصحة العامة. في المملكة العربية السعودية، يعتبر التسمم بالمبيدات الحشرية شائعا، حيث تُعد الكاربارمات والفوسفات العضوية من الأسباب الرئيسية.

****عرض الحالات**:**

تم توثيق أربع حالات تسمم حادة ناتجة عن تناول شطائر شاورما ملوثة تحتوي على الكاربوفيوران والبيفنثرين، وهي مزيج من الكاربارمات والبيريثرويد على التوالي. الأفراد المتضررون جميعهم أشقاء، وأظهروا درجات متفاوتة من الأعراض السمية، بما في ذلك ارتعاش العضلات، والإفرازات الزائدة، وضيق التنفس. على الرغم من شدة الأعراض، كان لدى ثلاثة من المرضى مستويات طبيعية أو منخفضة طبيعيًا من إنزيم الأسيتيل كولين إستريز، ويرجح أن يكون ذلك بسبب التباين الفردي، أو التأخير في إجراء الفحص، أو سوء التعامل مع العينات. أما المريض الرابع، فقد أظهر نشاطا منخفضا بشكل كبير لإنزيم الأسيتيل كولين إستريز، مما يشير إلى تثبيط شديد، وزادت الحالة تعقيدا بسبب التهاب البنكرياس، مما ساهم في تأخر التعافي. شملت التدخلات العلاجية السريرية المبكرة إعطاء الأتروبين، البراليدوكسيم، ومضاد التسمم الوشيقي بسبب الشك الأولي في وجود تسمم ناتج عن بكتيريا وشيقية. أكدت التحليلات السمومية تسمم الكاربوفيوران.

****الاستنتاجات**:**

تؤكد هذه الحالات على أهمية الاستشارة الفورية لمتخصصي طب السموم، وإجراء الفحوصات المناسبة، والتعامل الصحيح مع العينات لتوجيه العلاج في حالات التسمم بالمبيدات. تسلط هذه النتائج الضوء على الحاجة الماسة للالتزام بأنظمة منظمة الصحة العالمية ومراقبة استخدام المبيدات، بالإضافة إلى ممارسات صارمة لسلامة الغذاء لمنع تفشي الأمراض مستقبلاً..

## Introduction

**Figure 1:**
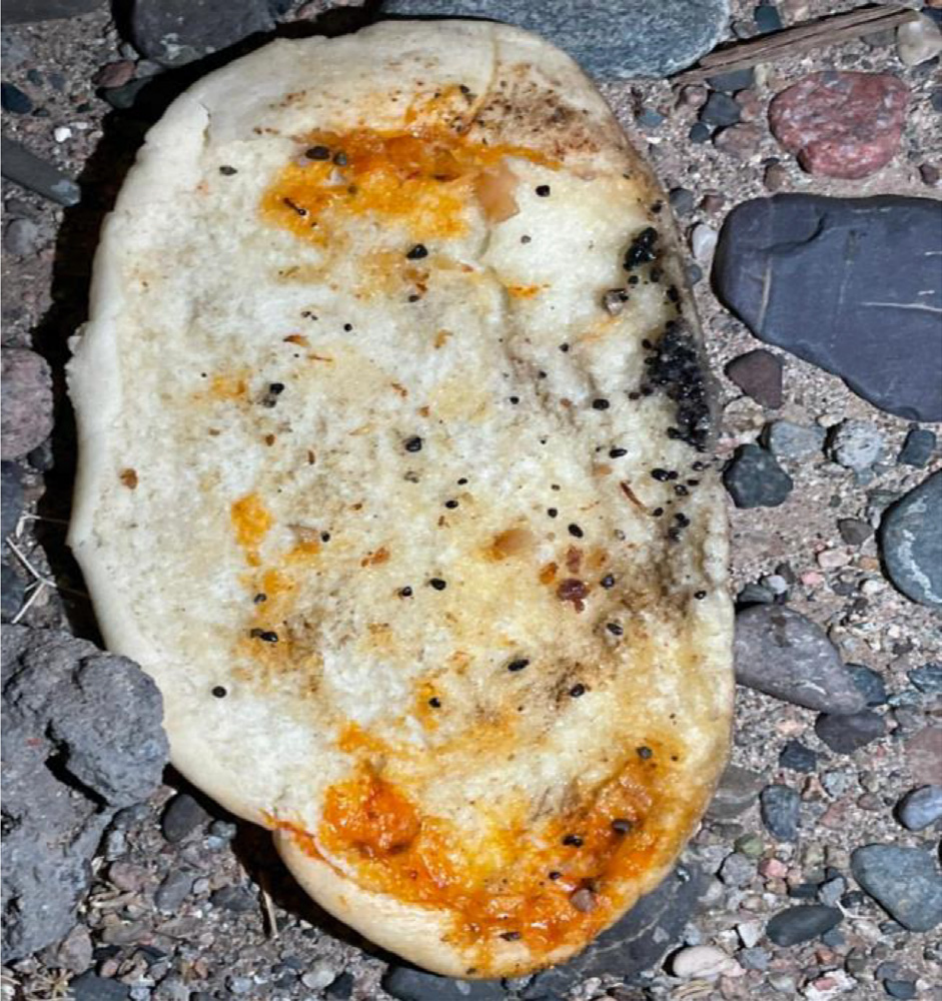
Black particles in contaminated sandwich bread.

**Figure 2:**
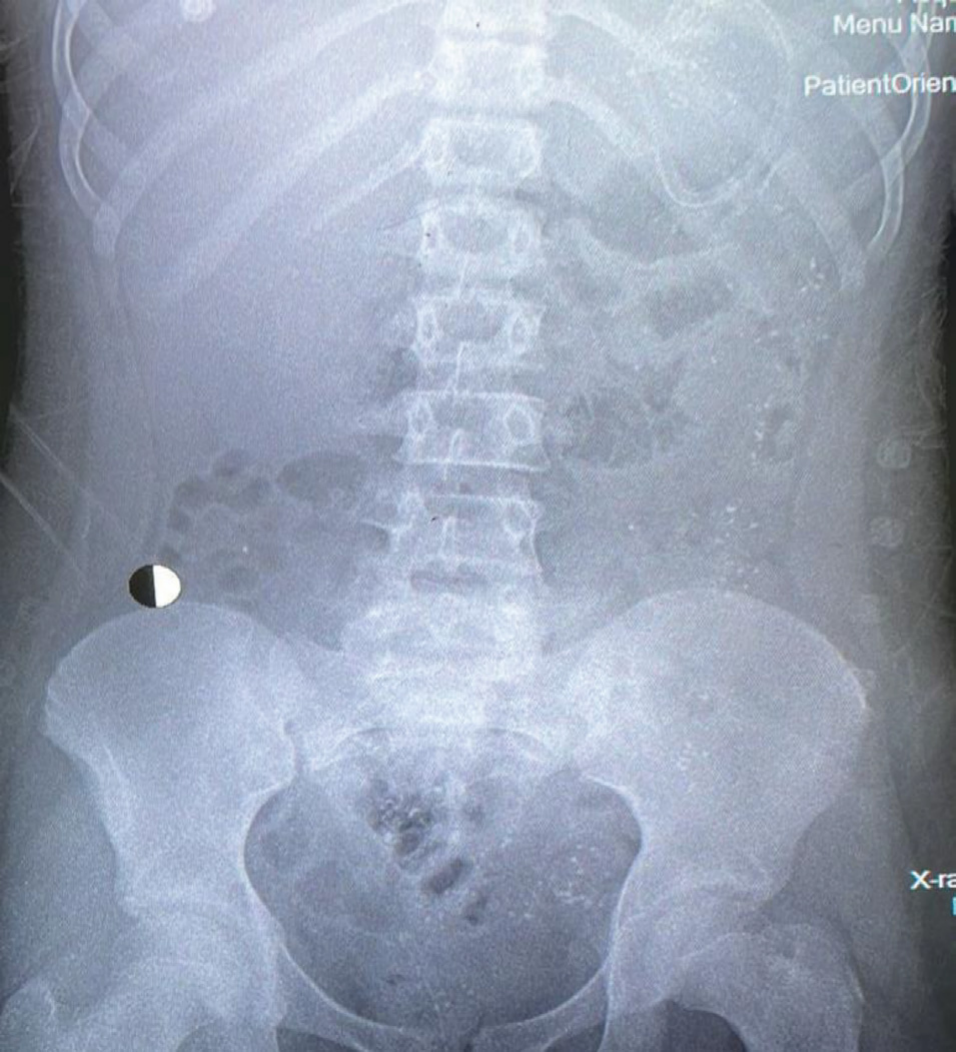
Abdominal X-ray showing scattered radio-opaque foreign bodies throughout the GI system.

**Figure 3:**
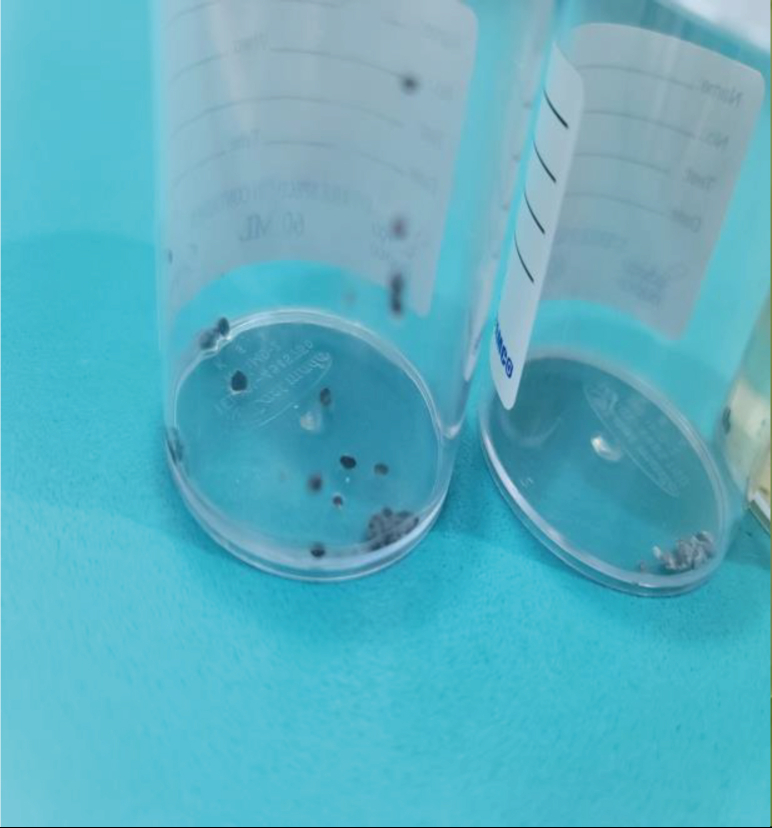
Gastric aspirate showing black particles.

**Figure 4:**
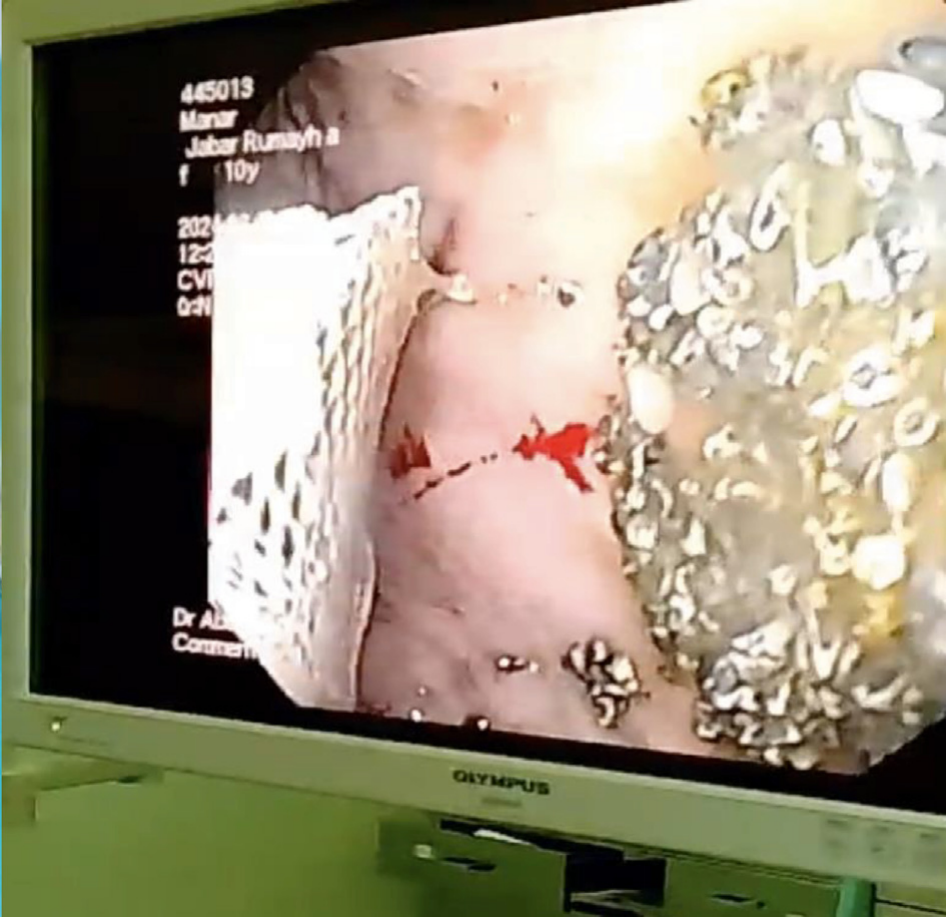
Upper endoscopy showing multiple minor erythematous lesions in the stomach, along with a bezoar of black particles.

Globally, pesticide self-poisoning results in approximately 258,234 deaths annually, accounting for 30 % of all suicides; adjusted estimates- taking into account underreporting in low- and middle- income countries- have increased this number to 371,594.^1^ Unintentional acute pesticide poisoning is a major global public health concern, which, in a recent systematic review, has been estimated to affect approximately 385 million people annually and result in 11,000 deaths, primarily among farmers and farmworkers.^2^ Food contamination with pesticides has been a persistent issue, particularly in regions with intensive agricultural practices. In December 2020, a pesticide-related outbreak in South India caused by triazophos contamination in household water supplies led to neurological symptoms in 400 individuals and resulted in 14 deaths.^3^ Similarly, in 2014, deliberate contamination of frozen food with malathion in Japan caused approximately 2800 cases of acute poisoning, thus emphasizing the need for stronger food safety measures.^4^ In KSA, the Al-Qassim region, recognized for its thriving agricultural sector, has been reported to have a high prevalence of pesticide-related poisoning cases. A recent 8-year retrospective study has indicated that 41.2 % of the 381 reported chemical poisoning cases in the region were attributed to insecticides and pesticides.^5^ Aluminum phosphide, a highly toxic insecticide and rodenticide, has been associated with numerous poisoning incidents in KSA; 82 % of related fatalities occurred in individuals under 20 years of age, primarily from accidental household exposure during fumigation.^6^ In a single-center, retrospective study conducted in the Eastern Region of KSA, 50 patients were exposed to organophosphates, primarily through the ingestion route (39 %), and 58 % of cases resulted from accidental exposure.^7^ In another single-center study in 82 patients, 62.2 % of cases were due to accidental exposure, whereas 30 % were suicide attempts. The most common routes of exposure were ingestion (55.41 %), skin contact (31.08 %), and inhalation (13.51 %). Most patients present with muscarinic effects, and nausea and vomiting occur in 62 % of cases.^8^ To our knowledge, documented cases of incidental exposure to a carbamate and pyrethroid mixture in KSA have been rare. In this report, we review four cases of carbofuran and bifenthrin poisoning in siblings after the ingestion of contaminated sandwiches. We further discuss the differential diagnosis and management of these cases (see Figure 1, Figure 2, Figure 3, Figure 4).

## Patient demographics and clinical presentation

### Patient 1: 11-year-old girl with epilepsy

An 11-year-old girl with a history of epilepsy managed with levetiracetam was transferred from a peripheral hospital to a tertiary care hospital after developing severe symptoms shortly after ingesting a shawarma sandwich containing hard black granules (approximately 2–5 mm in size) (Fig. 1). The family had eaten the shawarma at approximately 9:00 PM on July 29, 2024. Within 10 min of ingestion, the girl developed shivering, muscle twitching, severe anxiety, nausea, vomiting, and severe abdominal pain. Her condition deteriorated rapidly, and she showed excessive frothy oral secretions, respiratory difficulty, syncope, seizure-like activity characterized by an upward gaze, tongue fasciculation, and spasticity of the neck and extremities. Several of her family members who had consumed the same contaminated food, including her younger sibling and two adult family members, also exhibited symptoms to varying degrees.

She arrived at the peripheral hospital at 10:30 PM, where she presented with persistent vomiting, diarrhea, decreased consciousness, and upward gaze with tongue fasciculation. She showed pinpoint pupils. Her laboratory results revealed leukocytosis (white blood cell count [WBC] = 20 × 10⁹/L), hypokalemia (K = 2.9 mmol/L), and metabolic acidosis (pH = 7.20). Her initial treatments included intravenous fluids, antibiotics (ceftriaxone and metronidazole), and antiemetics. Given her deteriorating respiratory status, she was intubated and placed on mechanical ventilation before being transferred to the tertiary care hospital, where she arrived at 3:30 AM on July 30, 2024.

After arrival at the tertiary care hospital, she remained agitated, and showed a decreased level of consciousness, tachypnea, and respiratory distress. Copious frothy oral secretions and food particles were present in the endotracheal tube during suctioning. She had a fever (39.5 °C), tachycardia (HR = 140–160 bpm), and hypotension (BP = 90/45 mmHg) requiring vasopressor support. She exhibited pinpoint pupils, hyperreflexia, and continuous diarrhea. She remained on mechanical ventilation with decreased air entry and crepitations in the lungs. Chest X-ray revealed bilateral infiltrates, and laboratory tests indicated worsening leukocytosis (WBC = 22 × 10⁹/L), elevated creatine kinase (CPK = 970 U/L), and elevated serum amylase (701 U/L). On the basis of her clinical presentation and rapid deterioration, differential diagnoses of organophosphate poisoning, botulism, or other neurotoxic foodborne agents were considered. Toxicology testing, including serum acetylcholinesterase levels and analysis of the black granules, was initiated. Empiric therapy with atropine, pralidoxime, and botulinum antitoxin (heptavalent) were administered.

By the second day in the intensive care unit (ICU), her respiratory status remained critical, although her FiO_2_ requirement decreased from 80 % to 50 %. She continued to show pinpoint pupils with slow reactivity to light. Her gastrointestinal symptoms improved, and her diarrhea resolved. Her laboratory results revealed coagulopathy, with an elevated prothrombin time (PT = 22 s), activated partial thromboplastin time (APTT = 44 s), and INR of 1.7. She was treated with vitamin K and 6 units of fresh frozen plasma.

On the third day, the patient showed marked improvement, and her pupils were reactive to light (2–3 mm). Midazolam was discontinued, and her fentanyl dose was decreased. A chest and abdominal X-ray revealed small radiopaque particles in the stomach, which were partially removed through gastric aspiration. An upper gastrointestinal endoscopy confirmed the presence of black particles and multiple minor erythematous lesions in the stomach (Figure 2, Figure 3, Figure 4). Norepinephrine was discontinued, and the patient was prepared for extubation.

On the fourth day, the patient was successfully extubated and transitioned to a high-flow nasal cannula for post-extubation management. Her clinical condition continued to improve, and she was transferred to the pediatric medical ward for further recovery on the fifth day. She was discharged home in stable condition on the sixth day, with follow-up care arranged.

### Patient 2: girl 6 years 6 and months old

A girl 6 years 6 and months old, the younger sibling of patient 1, presented with milder symptoms after the ingestion of a contaminated shawarma sandwich. She vomited once within half an hour of eating the food but did not develop significant respiratory or neurological symptoms. However, she experienced marked fatigue rendering her unable to perform daily activities. Her vital signs were stable throughout the admission, and she showed no evidence of cranial nerve involvement, abnormal pupil responses, lacrimation, or salivation.

She arrived at the peripheral hospital at 10:30 PM and was closely monitored for 24 h, including hourly checks during the first day and every 2 h on the second day. Her laboratory findings, including renal and liver function tests, were normal. Toxicology tests, including blood samples, rectal swabs, and gastric aspirate, were sent for further analysis. Although her initial presentation was mild, she received botulinum antitoxin as a precaution, given the family history of multiple affected members. She was transferred to the tertiary care hospital, where she arrived at 3:30 AM. On the second day, her fatigue had improved, and she had regained her ability to perform daily activities. By the third day, she had returned to her baseline condition and showed normal clinical examination and laboratory findings. She remained hospitalized for 5 days and was discharged in stable condition.

### Patient 3: 18-year-old man

An 18-year-old man, the older brother of patient 1 and patient 2, who was previously healthy, was referred from the same peripheral hospital after developing gastrointestinal symptoms, including vomiting and diarrhea, 2 h after consuming the contaminated shawarma. He was seen at the peripheral hospital at 10:30 PM, where his condition deteriorated rapidly, including facial muscle fasciculations, excessive oral secretions, and respiratory difficulty, which were similar to those present in patient 1. After arrival at the tertiary care hospital, he had a blood pressure of 135/84 mmHg, heart rate of 118 bpm, oxygen saturation of 90 % on room air, and respiratory rate of 24 breaths per minute. Blood gas analysis revealed a pH of 7.24, PCO_2_ of 48 mmHg, and bicarbonate level of 18 mmol/L. An ECG revealed sinus tachycardia.

He became tachypneic and desaturated, showed progressive muscle weakness, and required intubation at 7:55 AM after receiving fentanyl and midazolam for sedation.

During the sedation vacation, he was conscious and able to follow commands, and showed strong motor function in all limbs. His pupils were bilaterally pinpoint, and a chest examination demonstrated good bilateral air entry without crackles. His chest X-ray findings were clear, showing no pulmonary infiltrates.

Laboratory tests revealed leukocytosis (WBC = 25 × 10⁹/L, 92 % neutrophils), a platelet count of 565 × 10⁹/L, hemoglobin level of 15 g/dL, creatinine level of 104 μmol/L, and urea level of 5.1 mmol/L. His coagulation study findings were within normal limits. Toxicology testing, including acetylcholinesterase levels and samples of serum and gastric fluid, were sent for analysis. The patient was treated with botulinum antitoxin (heptavalent), pralidoxime, and metronidazole (500 mg IV every 8 h). Full septic screening (blood, sputum, urine, and stool cultures) was also conducted.

After he was admitted to the ICU, sedation was discontinued for evaluation. He remained fully conscious, oriented, and obeying commands. His pupils remained bilaterally pinpoint, but he exhibited a good gag reflex and full limb strength. He was extubated after 2 h of continuous positive airway pressure (CPAP) support. By the next day, he was fully conscious, and had a Glasgow Coma Scale score of 15/15; he additionally tolerated oral feeding, passed normal bowel movements, and showed no neurological deficits. He was transferred to the general ward and remained stable throughout his hospital stay, with normalized WBC (11 × 10⁹/L) and platelet count (269 × 10⁹/L). He was discharged on August 7, 2024, in stable condition.

### Patient 4: 20-year-old woman

A 20-year-old woman, the eldest sibling of the family, with no prior medical history, also presented with vomiting and diarrhea 2 h after consuming contaminated shawarma with her family. She was seen at the peripheral hospital at 10:30 PM, and shortly thereafter developed facial muscle fasciculations, spasms, and excessive oral secretions, which were similar to those in her siblings. Her condition worsened rapidly, progressing to respiratory difficulty and descending muscle weakness. At the initial examination in the emergency department, she had a blood pressure of 145/81 mmHg, heart rate of 131 bpm, oxygen saturation of 88 % on room air, and respiratory rate of 26 breaths per minute. Venous blood gas analysis revealed a pH of 7.33, PCO_2_ of 24 mmHg, and bicarbonate level of 15 mmol/L. An ECG showed sinus tachycardia. She was intubated at 7:45 AM after sedation with fentanyl and midazolam, and placed on mechanical ventilation. She was transferred to the tertiary care hospital, where she arrived at 5:00 PM.

After later assessment during a sedation vacation, she was conscious and able to follow commands, and showed strong limb power and good neck flexion. Her pupils were bilaterally pinpoint, and a chest examination indicated adequate air entry without crackles. A chest X-ray revealed clear lungs with no infiltrates or signs of pulmonary edema.

Her laboratory investigations showed leukocytosis (WBC = 25 × 10⁹/L, 92 % neutrophils), platelet count of 855 × 10⁹/L, hemoglobin level of 11 g/dL, creatinine level of 102 μmol/L, and urea level of 5.7 mmol/L. Coagulation studies revealed an INR of 1.1, APTT of 33 s, and PT of 13 s. Toxicology testing and analysis of acetylcholinesterase levels were requested. She was treated with botulinum antitoxin (heptavalent), pralidoxime (1 g IV stat), and metronidazole (500 mg IV every 8 h). Full septic screening, including blood, sputum, urine, and stool cultures, was conducted.

In the ICU, sedation was discontinued, and she was extubated after 2 h on CPAP. She recovered quickly and showed no neurological deficits. By the third day of admission, she was fully conscious, had a Glasgow Coma Scale score of 15/15, and tolerated oral feeding. She exhibited no residual neurological symptoms, and her laboratory findings had normalized. She was transferred to the general ward and discharged in stable condition on day 9.

## Outbreak analysis and investigations timeline

After the arrival of family members to the peripheral hospital, because of suspicion for botulism, immediate action was taken. Blood and urine samples were collected from symptomatic family members, alongside gastric fluid samples, which were sent for toxicological and microbial testing. Simultaneously, the local public health department initiated an investigation into the restaurant that supplied the shawarma.

Public health authorities were notified, and an investigation team visited the restaurant where the contaminated shawarma was prepared. The authorities immediately closed the restaurant as a precautionary measure to prevent additional exposure. Environmental samples, including food remnants, water, and surface swabs, were collected from the restaurant for microbiological and toxicological analysis. Rectal, throat, and nasal samples were collected from food handlers for further investigation.

Preliminary results from the stool samples of two restaurant food handlers revealed contamination with *Klebsiella pneumoniae*, and one worker tested positive for *Enterobacter aerogenes*. *Escherichia coli* was detected in the restaurant's water supply. Additionally, Salmonella was identified in ranch sauce served with the shawarma. However, because these bacterial findings were not associated with the observed symptoms, the investigation focused on potential toxicological causes.

Toxicological analysis of the black particles in the sandwiches confirmed the presence of carbofuran at 2061.3 PPM, along with 3-hydroxycarbofuran (0.306 PPM) and bifenthrin. Carbofuran is a highly toxic carbamate pesticide known to inhibit cholinesterase enzymes, thus explaining the cholinergic symptoms in the affected individuals. Botulism was ruled out as the cause, because tests for *Clostridium botulinum* and botulinum toxin were negative.

Cholinesterase enzyme levels were measured in the affected individuals. One family member exhibited diminished cholinesterase activity (2438 U/L, normal range: 2879–12669 U/L), thereby confirming carbamate poisoning from carbofuran. The low-normal cholinesterase levels in the other family members indicated varying degrees of exposure but less severe poisoning. The presence of bifenthrin, a pyrethroid insecticide, contributed to the overall toxic load, although its effects were less significant than those of carbofuran.

The restaurant remained closed for 8 days while the public health authorities worked to mitigate further risks. Environmental cleaning and decontamination procedures were implemented, and the restaurant's water supply was treated to eliminate *E. coli* contamination. Food handlers underwent additional training in food safety protocols to prevent future incidents. After no further risk remained, the restaurant was allowed to reopen, and no additional cases of poisoning were reported.

### Discussion

This outbreak investigation underscores the critical importance of early recognition and prompt intervention in cases of foodborne poisoning, particularly when neurotoxic agents such as carbofuran and bifenthrin are involved. The rapid onset of symptoms in the affected family, after a recent botulism outbreak, presented substantial diagnostic challenges. Botulism typically has a delayed onset with respect to that of carbamate, and is characterized by dry mucous membranes and dilated pupils, whereas carbamate toxicity manifests more rapidly, with miosis and frothy secretions.^9^ Both conditions can result in respiratory distress, thus further complicating the clinical picture. Given the overlapping symptoms, and the temporal proximity to a botulism outbreak, differentiation required careful clinical judgment and toxicological analysis. Despite the diagnostic uncertainty, early administration of botulism antitoxin may be warranted in suspected cases, because this treatment can potentially improve outcomes in confirmed cases and prevent respiratory deterioration^9^. Given the similarity in neurological and respiratory presentation between botulism and carbamate poisoning, toxicological analysis was conducted for both botulinum toxin and pesticide exposure. Subsequent results confirmed that carbofuran and bifenthrin were the primary causative agents.

### Carbamate toxicity and mixture of carbamate and pyrethroids

Carbamate insecticides, introduced in the 1950s, were designed to achieve insecticide anticholinesterase activity with greater selectivity and lower toxicity to mammals than organophosphate insecticides. Carbofuran is a carbamate is classified by the World Health Organization (WHO) as a class Ib compound, denoting its “highly hazardous” status. Its toxicity arises from the reversible inhibition of acetylcholinesterase, and subsequent accumulation of acetylcholine at neuromuscular junctions and synapses.^10^ Consequently, overstimulation of cholinergic pathways produces the classical symptoms of cholinergic excess, including salivation, lacrimation, urination, defecation, gastrointestinal distress, and emesis (SLUDGE syndrome). Although most cases of carbamate poisoning are mild, severe cases typically occur after oral exposure, whether accidental or intentional^11^, ^12^, ^13^**.** In severe cases, poisoning may progress to muscle fasciculations, respiratory depression, and seizures due to central nervous system involvement.^14^^,^^15^ Carbamate poisoning usually resolves rapidly; however, when it is associated with pancreatitis, a rare complication, the hospital stay may be extended. In patient 1, the development of pancreatitis might have contributed to the extended hospital stay, in agreement with previous reports of carbofuran-induced complications.^16^

Bifenthrin, a pyrethroid type I insecticide, is classified by the WHO as a class II substance, indicating moderate hazard. It exerts its toxic effects by disrupting voltage-gated sodium channels in nerve cells, thereby leading to prolonged depolarization and overstimulation, and ultimately causing paralysis and death. Patients with bifenthrin poisoning typically show neurological symptoms, including fine tremors, prostration with coarse whole-body tremors, and elevated body temperature, and, in severe cases, may experience coma or death. The clinical presentation is often referred to as tremor syndrome (T-syndrome), which is characteristic of type I pyrethroid exposure.^17^^,^^18^

Carbamates do not enhance the acute toxicity of pyrethroids. Studies have shown that a combination of carbamates and pyrethroids does not increase the toxic effects; therefore, the mixture poses fewer acute toxicity concerns than other pesticide interactions.^14^

### Historical context of carbamate poisoning in KSA and the gulf region

Carbamate poisoning is relatively rare in KSA and the Gulf region, although sporadic reports have described cases of pesticide-related toxicity, most involving organophosphates. Carbamate is the second most frequently consumed pesticide after organophosphate in KSA.^19^ In a notable incident, four major outbreaks of acute poisoning with endrin, an organochlorine pesticide, occurred in Doha, Qatar, and Hofuf, KSA, between June and July 1967, affecting 874 people and causing 26 deaths. Investigations revealed that the victims had consumed bread made from flour contaminated with endrin, an organochlorine insecticide, which had leaked from containers stored above the flour during transport on two separate ships.^20^ The present cases are among the rare documented outbreaks of carbamate poisoning associated with food contamination in KSA, thus emphasizing the substantial risk of accidental exposure to carbamates in both urban and rural settings. The identification of carbofuran (2061.3 PPM) in the contaminated shawarma, along with bifenthrin, underscores the need for stricter regulation and monitoring of pesticide use, particularly in areas close to food production and distribution sites. Public health authorities should remain vigilant in tracking pesticide-associated illnesses and enforcing food safety regulations to prevent future outbreaks.

### Diagnostic challenges and public health implications

Given the patients’ symptoms and history of foodborne exposure, the initial suspicion for botulism was reasonable. However, after toxicological analysis excluded botulism and identified carbofuran and bifenthrin as the causative agents, the focus of treatment shifted to management of pesticide poisoning. Red blood cell and serum cholinesterase levels can both serve as proxies for neural tissue cholinesterase activity, but the former provide a more accurate reflection. In acute care settings, these assays are often unavailable in a timely manner to inform immediate management. Thus, emergency treatment should be based on clinical presentation. When available, test results require cautious interpretation, because of interindividual variability, the effects of comorbidities and medications, and the potential for laboratory errors.^21^ The acetylcholinesterase levels in patients 2, 3, and 4 were within normal or low-normal ranges despite their severe symptoms, probably because of individual variability in baseline enzyme levels or the timing of testing. Delayed testing might have allowed for partial recovery of enzyme activity, thus making the results less reflective of acute poisoning. Additionally, improper sample handling, such as inadequate storage at −20 °C or failure to maintain samples on ice during transport, might have caused spontaneous reactivation of the enzyme and led to false normal results. These factors complicate the interpretation of cholinesterase levels in patients who remain clinically symptomatic. In the first patient, the low acetylcholinesterase level indicated significant enzyme inhibition, which was compounded by the presence of pancreatitis, thereby contributing to the extended recovery time.

The public health investigation also revealed food safety failures at the implicated restaurant, where multiple sources of contamination—including Salmonella, *E. coli*, and *Klebsiella pneumoniae*—were identified. Although these bacterial contaminants were not directly responsible for the symptoms in the affected family, they highlight the broader food safety challenges faced by the restaurant industry. Prompt closure of the restaurant and environmental remediation were crucial in preventing further cases.

### Outcomes

Unlike organophosphates, carbamates bind reversibly to acetylcholinesterase, thus allowing for faster recovery of enzymatic function after the toxic agent is cleared. Despite this reversible nature, the treatment approach remains largely similar to that for organophosphate poisoning. Atropine is used to counteract muscarinic effects. Although existing evidence primarily supports rapid incremental dosing of atropine followed by a continuous infusion in decreasing mortality and morbidity in organophosphate poisoning, and in shortening hospital stay and recovery time, a similar approach may be applicable to carbamate poisoning.^22^ Atropine, according to American Heart Association recommendations, remains the cornerstone of treatment for both organophosphate and carbamate poisoning (class1A), and effectively mitigates parasympathetic overstimulation but does not reverse paralysis. The initial dose is doubled every 5 min until full atropinization is achieved, as indicated by a clear chest on auscultation, a heart rate above 80 beats per minute, and a systolic blood pressure greater than 80 mmHg. Early endotracheal intubation is recommended for patients with significant organophosphate poisoning, to improve clinical outcomes (class 1C). Benzodiazepines are used to manage seizures (class 1C), whereas oximes, which reactivate acetylcholinesterase, are more specific to organophosphate poisoning (class 2A). Despite limited evidence supporting oxime use in carbamate poisoning, oxime treatment should not be withheld when the specific toxin is unknown.^23^

Management of bifenthrin poisoning focuses on supportive care, with particular attention paid to neurological and respiratory symptoms to prevent severe complications.^18^^,^^24^

## Public health and environmental prevention

This outbreak underscores the critical need for comprehensive public health strategies to mitigate pesticide poisoning and adherence to WHO guidelines in classification and exposure to highly hazardous pesticides.^25^^,^^26^ Priorities include strengthening pesticide regulations, enforcing compliance, and mandating regular inspections of food production sites. Training food handlers and agricultural workers in pesticide safety and hygiene is essential. Public awareness campaigns should emphasize the risks of contamination and encourage early reporting of suspected cases. Implementing routine pesticide screening, environmental monitoring, and rapid diagnostic tools would improve early detection and response efforts. Effective collaboration among public health, agricultural, and environmental authorities is crucial in achieving a multidisciplinary approach to prevent further incidents.

In conclusion, the outbreak of carbofuran and bifenthrin poisoning highlights the importance of timely diagnosis, proper sample handling, and early intervention in pesticide-related poisoning. Variability in cholinesterase levels complicates diagnosis, thereby emphasizing the need for clinical judgment. Strengthened food safety regulations and pesticide monitoring are crucial for preventing similar incidents.

## Declaration of Generative AI and AI-assisted technologies in the writing process

During the preparation of this work, I, Musa S. Alfaifi (principal author) used ChatGPT, an AI language model developed by OpenAI, to assist in writing and improving the language of this research manuscript. The AI tool was used primarily for language enhancement, grammatical correction, and stylistic improvement, whereas all content, concepts, ideas, and scientific analysis remain my own. Any information, interpretations, and conclusions presented in the manuscript are solely the product of my work, and ChatGPT was used strictly as a tool to enhance clarity and coherence in language. After using ChatGPT, I reviewed and edited the content as needed and take full responsibility for the content of the publication.

## Source of funding

This study had no funding sources.

## Author contributions

Conceptualization: MSA.

Data Collection: Nafaa Abdulalem, Hussein A. Albogami, Hamoud M. Alrougi, Adel Altowairqi, Rayyan Saqah, Maged Asaad Hamoud, Hajer. F. Ali, Zohair Al Aseri.

Manuscript Writing—Original Draft: MSA.

Review & Editing: Abrar Alasmari, Bader Alyahya, Zohair Al Aseri, Hussein A. Albogami, Hamoud M. Alrougi.

Supervision: Abdullah M. Assiri, Zohair Al Aseri.

All authors have critically reviewed and approved the final draft and are responsible for the content and similarity index of the manuscript.
